# P084: Analysis of therapeutic efficacies of amodiaquine-arstesunate and artemether-lumefantrine for treatment of uncomplicated falciparum malaria in Burkina Faso five years after their implementation

**DOI:** 10.1186/2047-2994-2-S1-P84

**Published:** 2013-06-20

**Authors:** FW Nikiema, I Zongo, F Some, J-B Ouedraogo, L Penali

**Affiliations:** 1 Health Research, Institut de Recherche en Science de la Sante de Bobo Dioulasso, Bobo Dioulasso, Burkina Faso; 2 Data management, WorldWide Antimalarial Resistance Network, West Africa , Dakar, Senegal

## Introduction

Since 2005, Burkina Faso adopted artesunate plus amodiaquine (ASAQ) and artemether-lumefantrine (AL) as first-line treatment for uncomplicated malaria. Despite improvement in that treatment, malaria remains the first cause of morbidity and mortality in the country.

## Objectives

This study aimed to analyze the therapeutic efficacies of ASAQ and AL for the treatment of uncomplicated *falciparum* malaria in Burkina Faso five years after their adoption.

## Methods

Per-protocol individual data from four randomized clinical trials supported by IRSS-DRO Bobo Dioulasso in 2006, 2008, 2009 and 2010, including 1076 patients with uncomplicated *P. falciparum* malaria, treated with the recommended regimen of AL or ASAQ, were analyzed according to WWARN analytical methods. Patients benefited from a clinical and biological 28-day follow-up and performed on days 2, 3, 7, 14 and 28 to evaluate clinical and parasitological outcomes. Treatment failures have been corrected by PCR.

## Results

Using WWARN analytical methods, the unadjusted Kaplan-Meier survival estimates are 76.4% (95% CI (72.5-79.8)) in the AL group (N=544) and 87.1% (95% CI (83.9-89.7)) in the ASAQ group (N=532). After PCR correction, AL was less efficacious than ASAQ respectively 95.8% (95% CI (93.6-97.3)) vs 98.2% (95% CI (96.6-99.1)); OR=0,486 (95% CI (0.217-1.089). There was no significant correlation between the occurrence of recrudescent at day 28 end-point and study year in two groups (coefficient<0).

## Conclusion

AL and ASAQ remain effective as treatment for uncomplicated malaria according to WHO recommendations, though AL was inferior in preventing recrudescent for 28-day follow-up.

## Disclosure of interest

None declared.

